# Resurrecting a subgenus to genus: molecular phylogeny of *Euphyllia* and *Fimbriaphyllia* (order Scleractinia; family Euphyllidae; clade V)

**DOI:** 10.7717/peerj.4074

**Published:** 2017-12-04

**Authors:** Katrina S. Luzon, Mei-Fang Lin, Ma. Carmen A. Ablan Lagman, Wilfredo Roehl Y. Licuanan, Chaolun Allen Chen

**Affiliations:** 1 Biology Department, De La Salle University, Manila, Philippines; 2 Shields Ocean Research (SHORE) Center, De La Salle University, Manila, Philippines; 3 The Marine Science Institute, University of the Philippines, Quezon City, Philippines; 4 Philippine Council for Agriculture, Aquatic and Natural Resources Research and Development (PCAARRD), Los Baños, Laguna, Philippines; 5 Biodiversity Research Center, Academia Sinica, Taipei, Taiwan; 6 Department of Molecular and Cell Biology, James Cook University, Townsville, Australia; 7 Evolutionary Neurobiology Unit, Okinawa Institute of Science and Technology Graduate University, Okinawa, Japan; 8 Center for Natural Sciences and Environmental Research (CENSER), De La Salle University, Manila, Philippines; 9 Taiwan International Graduate Program-Biodiversity, Academia Sinica, Taipei, Taiwan; 10 Institute of Oceanography, National Taiwan University, Taipei, Taiwan

**Keywords:** Phylogeny, Taxonomy and systematics, Integrative systematics

## Abstract

**Background:**

The corallum is crucial in building coral reefs and in diagnosing systematic relationships in the order Scleractinia. However, molecular phylogenetic analyses revealed a paraphyly in a majority of traditional families and genera among Scleractinia showing that other biological attributes of the coral, such as polyp morphology and reproductive traits, are underutilized. Among scleractinian genera, the *Euphyllia*, with nine nominal species in the Indo-Pacific region, is one of the groups that await phylogenetic resolution. Multiple genetic markers were used to construct the phylogeny of six *Euphyllia* species, namely *E. ancora, E. divisa, E. glabrescens, E. paraancora, E. paradivisa,* and *E. yaeyamaensis.* The phylogeny guided the inferences on the contributions of the colony structure, polyp morphology, and life history traits to the systematics of the largest genus in Euphyllidae (clade V) and, by extension, to the rest of clade V.

**Results:**

Analyses of cytochrome oxidase 1 (*cox1*), cytochrome b (*cytb*), and β-tubulin genes of 36 colonies representing *Euphyllia* and a confamilial species, *Galaxea fascicularis,* reveal two distinct groups in the *Euphyllia* that originated from different ancestors. *Euphyllia glabrescens* formed a separate group. *Euphyllia ancora, E. divisa, E. paraancora, E. paradivisa,* and *E. yaeyamaensis* clustered together and diverged from the same ancestor as *G. fascicularis.* The 3′-end of the *cox1* gene of *Euphyllia* was able to distinguish morphospecies.

**Discussion:**

Species of *Euphyllia* were traditionally classified into two subgenera, *Euphyllia* and *Fimbriaphyllia,* which represented a dichotomy on colony structure. The paraphyletic groups retained the original members of the subgenera providing a strong basis for recognizing *Fimbriaphyllia* as a genus. However, colony structure was found to be a convergent trait between *Euphyllia* and *Fimbriaphyllia,* while polyp shape and length, sexuality, and reproductive mode defined the dichotomy better. Species in a genus are distinguished by combining polyp morphology and colony form. The cluster of *E. glabrescens* of the *Euphyllia* group is a hermaphroditic brooder with long, tubular tentacles with knob-like tips, and a phaceloid colony structure. The *Fimbriaphyllia* group, with *F. paraancora, F. paradivisa, F. ancora, F. divisa,* and *F. yaeyamaensis,* are gonochoric broadcast spawners with short polyps, mixed types of tentacle shapes, and a phaceloid or flabello-meandroid skeleton. Soft-tissue morphology of *G. fascicularis* and *Ctenella chagius* were found to be consistent with the dichotomy.

**Conclusions:**

The paraphyly of the original members of the previous subgenera justify recognizing *Fimbriaphyllia* as a genus. The integrated approach demonstrates that combining polyp features, reproductive traits, and skeletal morphology is of high systematic value not just to *Euphyllia* and *Fimbriaphyllia* but also to clade V; thus, laying the groundwork for resolving the phylogeny of clade V.

## Introduction

Systematics of the Scleractinia are traditionally based on features of the skeleton (also called the corallum) (Dana, 1846; Edwards & Haime, 1857; Edwards & Haime, 1860; Vaughan & Wells, 1943; Veron & Pichon, 1980; Veron, 2000). Despite the convenience of relying on the corallum, the skeleton is plagued with taxonomic ambiguities brought about by plasticity and convergence, which is a weakness in the traditional systematics of the Scleractinia. Lang (1984) proposed searching for other biological attributes for identification such as polyp or soft-tissue morphology and anatomy, mode of reproduction, behaviors, and ecological and physiological aspects of corals. These traits or a combination of any number of them are thought to have greater systematic value than adhering strictly to skeletal features (Daly, Fautin & Cappola, 2003). Recent advancements in molecular phylogenetic construction of the evolutionary history of scleractinian corals echo this proposal along with proposed major deviations from traditional classification schemes. The first deviation was the discovery of two major lineages within the order, now referred to as the robust and complex clades (Romano & Palumbi, 1996; Romano & Palumbi, 1997; Romano & Cairns, 2000; Chen, Wallace & Wolstenholme, 2002; Fukami et al., 2008; Huang et al., 2009; Kitahara et al., 2010; Okubo, 2016). The second deviation showed that paraphyly was found in 11 traditional families of the Scleractinia (Fukami et al., 2008; Huang et al., 2009; Huang et al., 2011; Huang et al., 2014a; Huang et al., 2014b; Arrigoni et al., 2012). These findings further suggested that skeletal features (colony formation, corallite diameter, and characteristics of the septa and costae) that are widely used in identifying species of corals are not fully reflective of evolutionary relationships within families and even between conspecific populations from the Atlantic, Pacific, and Indian Oceans (Chen et al., 1995; Romano & Palumbi, 1996; Romano & Palumbi, 1997; Romano & Cairns, 2000; Fukami et al., 2004; Fukami et al., 2008; Budd & Stolarski, 2009; Kitahara et al., 2010; Kerr, 2005; Arrigoni et al., 2012; Arrigoni et al., 2014a; Arrigoni et al., 2014b; Arrigoni et al., 2016). These deviations have challenged systematists to reexamine phylogenetic groupings in contrast with the traditional families and to discern and propose characteristics, apart from the skeleton or other aspects of the skeleton, that are systematically informative and diagnostic of species in the new groupings. This integrated approach to systematics has led to remarkable resolutions in some scleractinian families. The Family Acroporidae, for example, one of the largest families in the Scleractinia, traditionally classified *Acropora* and *Isopora* as the two major subgenera in the family. Morphological characteristics and reproductive traits that were found to reflect the phylogenetic relationships in the family led to the recognition of *Acropora* and *Isopora* as independent genera (Fukami, Omori & Hatta, 2000; Van Oppen et al., 2001; Wallace et al., 2007). The characteristics and the attributes that were identified through the integrated approach have been demonstrated to be operationally useful in subsequent classifications in the group as in the example of *Isopora togianensis* (Wallace et al., 2007). Currently, the Faviidae, Merulinidae, Pectiniidae, Trachyphyllidae, and a new family, Lobophyllidae, are nearly completely phylogenetically resolved and revised (Huang et al., 2009; Huang et al., 2011; Huang et al., 2014a; Huang et al., 2014b; Arrigoni et al., 2012; Arrigoni et al., 2014a), but other groups, such as the family Euphyllidae (clade V), still lack their respective phylogenetic investigations.

The family Euphyllidae originally had 14 species classified into five genera namely the *Euphyllia, Catalaphyllia, Plerogyra, Physogyra,* and *Nemenzophyllia* (Veron, 2000). The *Euphyllia,* the largest genus in the family, was classified in earlier systematic schemes under the subfamily Eusmilinae of the Family Caryophyllidae (Vaughan & Wells, 1943; Veron & Pichon, 1980). Veron & Pichon (1980) recognized a dichotomy in the genus *Euphyllia* that is based on colony structure and represented this dichotomy as the subgenera *Euphyllia* and *Fimbriaphyllia.* The subgenus *Euphyllia* included species with a phaceloid growth form, which listed *E. glabrescens* and *E. cristata* in the group*.* The subgenus *Fimbriaphyllia*, on the other hand, included species with a flabello-meandroid growth form namely, *E. ancora* and *E. divisa.* The subgenera were eventually synonymized as *Euphyllia*; however, Veron (2000) retained the dichotomy based on colony structure as more species were discovered and classified under the genus *Euphyllia* (Veron, 1990)*.* Eight species were then recognized and classified into two groups based on colony structure. One group, with phaceloid skeletons, included *E. glabrescens, E. cristata, E. paraancora, E. paradivisa,* and *E. paraglabrescens*; and the other group, with flabello-meandroid skeletons, included *E. yaeyamaenesis, E. divisa,* and *E. ancora* (Veron, 2000; Table 1)*.* A ninth species, *E. baliensis,* with a phaceloid colony structure, was recently discovered from Bali, Indonesia (Turak, Devantier & Erdmann, 2012). Corallite features and tentacle shapes are both considered when classifying *Euphyllia* because dried skeletons that have the same colony structure are difficult to tell apart without viewing the live form (Veron & Pichon, 1980; Veron, 2000). Yet, an overlap in skeletal and tissue characteristics was also observed between species from different groups (Table 1). Recently, reproductive traits were recognized as an excellent guide for systematic affinities among the Scleractinia (Kerr, Baird & Hughes, 2011; Baird, Guest & Willis, 2009). For example, the phaceloid species, *E. glabrescens,* is hermaphroditic with a reproductive mode of brooding, while the flabello-meandroid species, *E. ancora* and *E. divisa,* are gonochoric with a reproductive mode of broadcast spawning (Table 1; Baird, Guest & Willis, 2009).

Scleractinian phylogeny constructed using mitochondrial cytochrome oxidase I (*cox1*)*,* cytochrome oxidase *b* (*cytb*)*,* and nuclear β-tubulin showed that the family Euphyllidae is polyphyletic, with its members diverging from each other into the robust and complex clades (Fukami et al., 2008). *Physogyra lichtensteni* and *Plerogyra sinuosa* were clustered under clade XVI of the robust clade together with *Plesiastrea versipora* (Faviidae) and *Blastomussa wellsi* (Mussidae) (Fukami et al., 2008; Arrigoni et al., 2012)*.* The genus *Euphyllia,* on the other hand, had some of its members cluster under clade V, which was still designated by Fukami et al. (2008) as the family Euphyllidae, of the complex clade. A paraphyletic pattern in the genus was already observed when *E. glabrescens* grouped with *Ctenella chagius* (Meandrinidae), and *E. ancora* and *E. divisa* grouped with *Galaxea fascicularis* (Oculinidae) (Fukami et al., 2008).

In this study, we utilized multiple genetic markers to construct the phylogeny of *Euphyllia* collected from the Philippines and Taiwan, including *E. paraancora, E. paradivisa, E. yaeyamaensis, E. divisa, E. ancora,* and *E. glabrescens.* The former three species do not have a clear phylogenetic status and/or have not yet been analyzed from the molecular perspective before. The phylogeny was used in examining the internal relationships in *Euphyllia* and in identifying the relevant morphological and reproductive traits to the systematics of the genus. The *Euphyllia* is currently the largest genus in clade V and the inferred traits from the internal phylogeny was extended to infer the external relationships of the genus to the other members of the clade; thus, laying the groundwork for the resolution of clade V.

## Methodology

### Sample collection and specimen identification

In total, 36 colonies representing six species of *Euphyllia* and *G. fascicularis* were collected and sampled from two areas of western Luzon, the Philippines and in Kenting National Park, Taiwan (Table S1). These two locations in the Philippines included Talim Bay, Lian, Batangas and Bolinao, Pangasinan; the latter is where Veron (1990) first found and described *E. paraancora*. All coral samples from the Philippines were collected through permissions granted by the Bureau of Fisheries and Aquatic Resources (BFAR) permit number FBP-0021-08. The sample from Taiwan was collected through permissions granted by the Kenting National Park Headquarters as part of a long-term monitoring program (Project 673202-LTER). All specimens were identified in the field using Veron (1990), Veron (2000), and Veron & Pichon (1980). Specimens were photographed underwater with a Canon A710 camera. After collection and tissue sampling, coral colonies were bleached, and skeletons were examined and kept at the Coral Museum of The Marine Science Institute, University of the Philippines.

**Table 1:** Characteristics of *Euphyllia*, *Galaxea*, and *Ctenella*. Colony structure, corallites, tentacle morph, sexuality, and reproductive mode of *Euphyllia* spp, *Galaxea* sp., and *Ctenella chagius*. Data were modified from Veron & Pichon (1980)^a^, Veron (2000)^b^, Sheppard, Dinesen & Drew (1983)^c^, and Baird, Guest & Willis (2009)^d^. Species names listed in Group 1 and Group 2 that are in bold are the original members of the subgenera *Euphyllia* and *Fimbriaphyllia* in groups 1 and 2 respectively.

**Species**^a,b,c^	**Colony structure**^a,b^	**Corallites**^a,b,c^	**Tentacle morph**^b,c^	**Sexuality**^d^	**Reproductive mode**^d^
**Group 1**					
***E. glabrescens***	Phaceloid	First and second order septa plunge steeply near the centre of the corallite. Columella is absent.	Long tubular tentacles with knob-like tips	Hermaphroditic	Brooding
E. cristata^*^	Phaceloid	First and second order septa plunge steeply near the centre of the corallite. Columella is absent.	Long tubular tentacles with knob-like tips	Unrecorded	Unrecorded
E. paraglabrescens^*^	Phaceloid	Skeletons are almost identical to those of *E. glabrescens.*	Tentacles are short and bubble-like	Unrecorded	Unrecorded
*E. paraancora*	Phaceloid	Skeletons are like those of *E. glabrescens* with corallites 20–40 mm in diameter.	Tentacle tips form concentric circles and are shaped like an anchor, bean, or a kidney	Unrecorded	Unrecorded
*E. paradivisa*	Phaceloid	Skeletons are like those of *E. glabrescens.*	Branching tentacles almost identical to those of *E. divisa*	Unrecorded	Unrecorded
E. baliensis^*^,^#^	Phaceloid	Corallites are sub-circular, with non-budding corallites averaging 3.1 mm diameter and ranging from 2–4.1 mm, with very thin walls	Tentacles are shaped like an anchor, kidney, or hammer at their tips, occasionally with additional smaller bulbous protuberances, the latter resembling mittens or gloves.	Unrecorded	Unrecorded
**Group 2**					
***E. divisa***	Flabello-meandroid	There are three orders of septa, which are exsert and plunge near the valley centre. Columella is absent.	Polyps have large tubular tentacles with smaller tubular branches. All branches have knob-like tips	Gonochronic	Broadcast spawning
***E. ancora***	Flabello-meandroid	Colonies have the same skeletal structure as *E. divisa*	Polyps have large tubular tentacles with few or no branchlets but with tips shaped like an anchor, bean, kidney, hammer, or a letter ‘T’	Gonochronic	Broadcast spawning
*E. yaeyamaensis*	Phaceloid/ Flabello-meandroid (with short valleys)	Septa occur in three orders and are usually compact. Columella is absent.	Tentacles are short and fleshy and covered with short uniform branchlets, each with a terminal knob	Unrecorded	Unrecorded
**Group 3**					
*Galaxea fasicularis*	Plocoid	Corallites are of mixed sizes, usually less than 10 mm diameter with numerous septa reaching the corallite centre. Columella is absent.	Tubular tentacles with white tips, usually extended during the day	Pseudogynodiecious	Broadcast spawning
**Group 4**					
*Ctenella chagius*	Meandroid	Two orders of septa with thick primary septa. Septa have small denticles and minute spinules. Columella is present.	Tubular tentacles extended during the day	Unrecorded	Unrecorded

**Notes.**

* not analyzed in this study.

# species described by Turak, Devantier & Erdmann (2012).

### DNA extraction and purification

A minimum of 1 cm^3^ of a coral colony, includes both tissue and skeleton, was pruned off a sample with an orthopedic bone cutter. The pruned tissue with the skeleton was stored in pre-labeled 15 ml conical tubes containing CHAOS (Chaotropic solution: 4 M guanidine thiocyanate, 0.5% N-lauroyl sarcosine sodium salt, 25 mM Tris at pH 8, and 0.1 M 2-mercaptoethanol) (Fukami et al., 2004) that was 3∼5-times the volume of the sample taken (i.e., 1 cm^3^ of tissue entailed 3∼5 mL of CHAOS). The tubes were kept in the dark for five days at room temperature. After the five-day period, DNA was obtained through the standard phenol/chloroform purification method with phenol extraction buffer (100 mM Tris-Cl at pH 8, 10 mM EDTA, and 0.1% sodium dodecylsulfate) (Chen & Yu, 2000; Chen et al., 2000). Purified DNA was quantified through spectrophotometry with Nanodrop 1000 by Thermo Fisher Scientific (Waltham, MA, USA) and through agarose gel electrophoresis (0.5% Seakem® LE agarose; Lonza, Basel, CH). The molecular weight of each sample was estimated using the lambda ladder of Protech Technology Enterprise (Taipei, Taiwan).

### DNA sequencing

The *cytb* gene was amplified using newly developed primer pairs that were designed especially for *Euphyllia.* The *cytb* primer pairs have the following sequences: Eu4500F-1 (5′-CTG TCT AGT TTG GGA GTT AA-3′) and Eu4500R (5′-ATC ACT CAG GCT GAA TAT GC-3′) (set 1); and Eu4500F (5′-GAC AGA TGT TGT GCA ATG AG-3′) and Eu4500R-1 (5′-AAT AAG GCT ACC ATA AGC C-3′) (set 2). The expected product sizes of the amplicon for each pair were 1.0 and 1.5 kb, respectively. Two sets of primer pairs, developed by Lin et al. (2011), that amplified the 3′-end region of scleractinian *cox1* were utilized: Cs-F17-a (5′-CCA TAA CCA TGC TTT TAA CGG ATA-3′) and Cs-R17-a (5′-TGC TAA TAC AAC TCC AGT CAA ACC-3′); and Cs-F18 (5′-GGA CAC AAG AGC ATA TTT TAC TG- 3′) and Cs-R18 (5′-CTA CTT ACG GAA TCT CGT TTG A-3′). The expected product sizes of the amplicon from each pair were 1,400 and 950 bp, respectively. The β-tubulin gene was amplified using a primer pair developed by Fukami et al. (2004): forward (5′-GCA TGG GAA CGC TCC TTA TTT-3′) and reverse (5′-ACA TCT GTT GAG TGA GTT CTG-3′). The β-tubulin gene was expected to yield multiple gene copies with base pair lengths of 0.6, 1.5, and 2.0 kb (Fukami et al., 2004).

A polymerase chain reaction (PCR) was carried out in 50 µl reactions with either Pro-taq polymerase (Protech Technology Enterprise, Taipei, Taiwan) or Invitrogen™ taq-polymerase (Thermo Fisher Scientific, Waltham, MA, USA). Amplifications performed with the Invitrogen taq-polymerase contained a final concentration of the following: 2.0 mM of each base of dNTP, 3.0 mM of MgCl_2_, 1× Invitrogen buffer (200 mM Tris-Cl at pH 8.4), 0.4 µM of primers, 1 unit of taq-polymerase, 2% DMSO, and at least 20 ng/µl of the DNA template. Amplifications performed with the Protech taq-polymerase, on the other hand, contained a final concentration of 2.0 mM of each base of dNTP, 1× Protech buffer (with MgCl_2_), 0.4 µM of the primers, 5 units of the taq-polymerase, 2% DMSO, and at least 20 ng/µl of the DNA template. The PCR was carried out with a PxE Thermal Cycler by Thermo Fisher Scientific (Waltham, MA, USA). Amplification of the mitochondrial genes began with an initial denaturation temperature of 95 °C for 3 min, followed by 30 cycles of denaturation at 94 °C for 30 s, annealing at 50 °C for 1 min, and elongation at 72 °C for 90 s, with one final extension step at 72 °C for 10 min. The β-tubulin gene was amplified with an initial denaturation step at 94 °C for 2 min; followed by 30 cycles of denaturation at 94 °C for 45 s, annealing at 60 °C for 45 s, and elongation at 72 °C for 90 s, with one final extension step at 72 °C for 5 min. Among multiple copies of the β-tubulin gene of *Euphyllia,* the 600-bp band was selected for cloning because it was found to be present in all samples. Ligation and transformation of the β-tubulin amplicons was performed with a pGEM®-T Easy Vector System kit from Promega (Madison, WI, USA). Transformants were cultured in LB/ampicillin/IPTG/X-Gal plates, and five pure-white colonies were selected per sample. PCR products from all markers were purified with the PCR-M™ Clean-Up System of Viogene (New Taipei City, Taiwan) prior to sequencing.

### Phylogenetic analyses

Contiguous sequences (contigs) were assembled and annotated with Genious Pro vers. 4.6.1 software (Drummond et al., 2011). Each of the contigs was searched for in the database of NCBI BLAST to determine if the sequences matched a member of the Scleractinia. The search was also utilized to check for the direction of the sequences in the scleractinian sequences that they matched, especially with the clones of the β-tubulin gene. Sequences of a gene were then aligned using the CLUSTAL W plug-in of the software MEGA 5 (Tamura et al., 2011). *Cytb* and *cox1* segments from the complete mitochondrial genome of *E. ancora* (GenBank accession nos. NC015641 (*cytb*) and JF825139 (*cox1*); Lin et al., 2011) were used to guide the mitochondrial gene alignments*.* Available sequences of *E. glabrescens, E. ancora, E. divisa, G. fascicularis, C. chagius*, and other scleractinians were also gathered from the NCBI database.

Phylogenetic trees were then generated for each set of genes and combined mitochondrial genes (*cox1* and *cytb*) using the Bayesian inference (BI) and maximum likelihood (ML) methods. The Bayesian inference was performed with the software Mr. Bayes 3.2.2 (Ronquist et al., 2012). Two runs were carried out with four Markov chains in 2 million generations, and the first 25% of trees were discarded as burn-in. Convergence of the BI analyses was determined by the average standard deviation (SD) of split frequencies (<0.01). ML trees were generated with 1,000 bootstrap replicates in MEGA versions 5 and 6 (Tamura et al., 2011). The best-fit models of evolution for the BI and ML analyses were obtained with jModeltest software (Guindon & Gascuel, 2003; Posada, 2008) (Table S2). The best-suited model was determined with a 95% confidence level using the Akaike information criterion (AIC) (Posada & Buckley, 2004).

## Results

**Figure 1:**
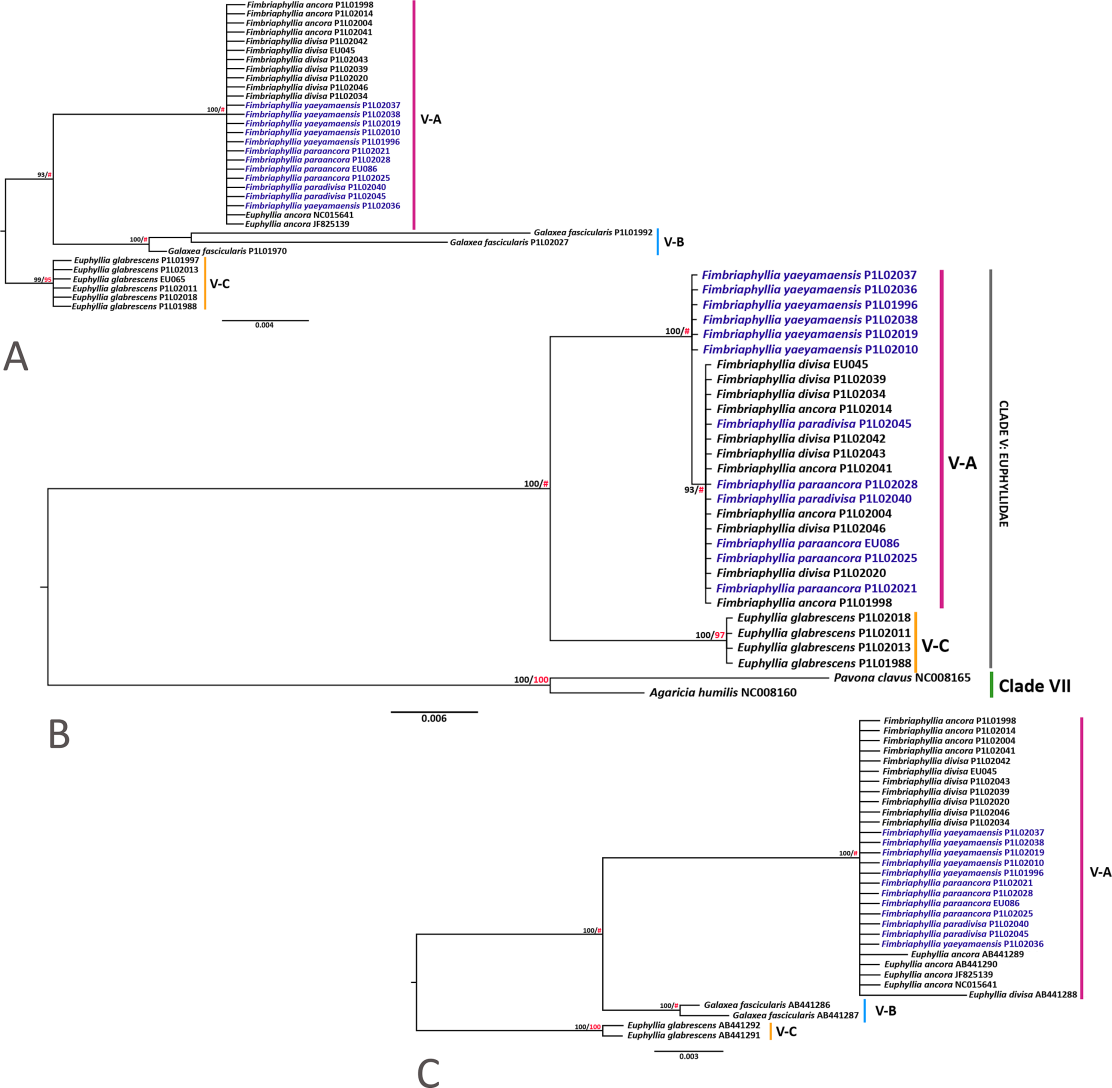
Phylogenetic trees of the *cox1, cytb,* and the combined *cox1* and *cytb* genes with sequences from clade VII as an outgroup*.* The phylogenetic trees of the (A) *cox1* gene*;* (B) combined *cox1* and *cytb* genes; and (C) *cytb* gene.** Bootstrap values of BI (black)/ML (red) are indicated before the nodes of the clusters. # indicates a difference in topologies between the BI and ML gene trees. Species names in blue font were analyzed herein for the first time. Distinct clusters in the tree are distinguished with vertical lines and labeled chronologically.

**Figure 2:**
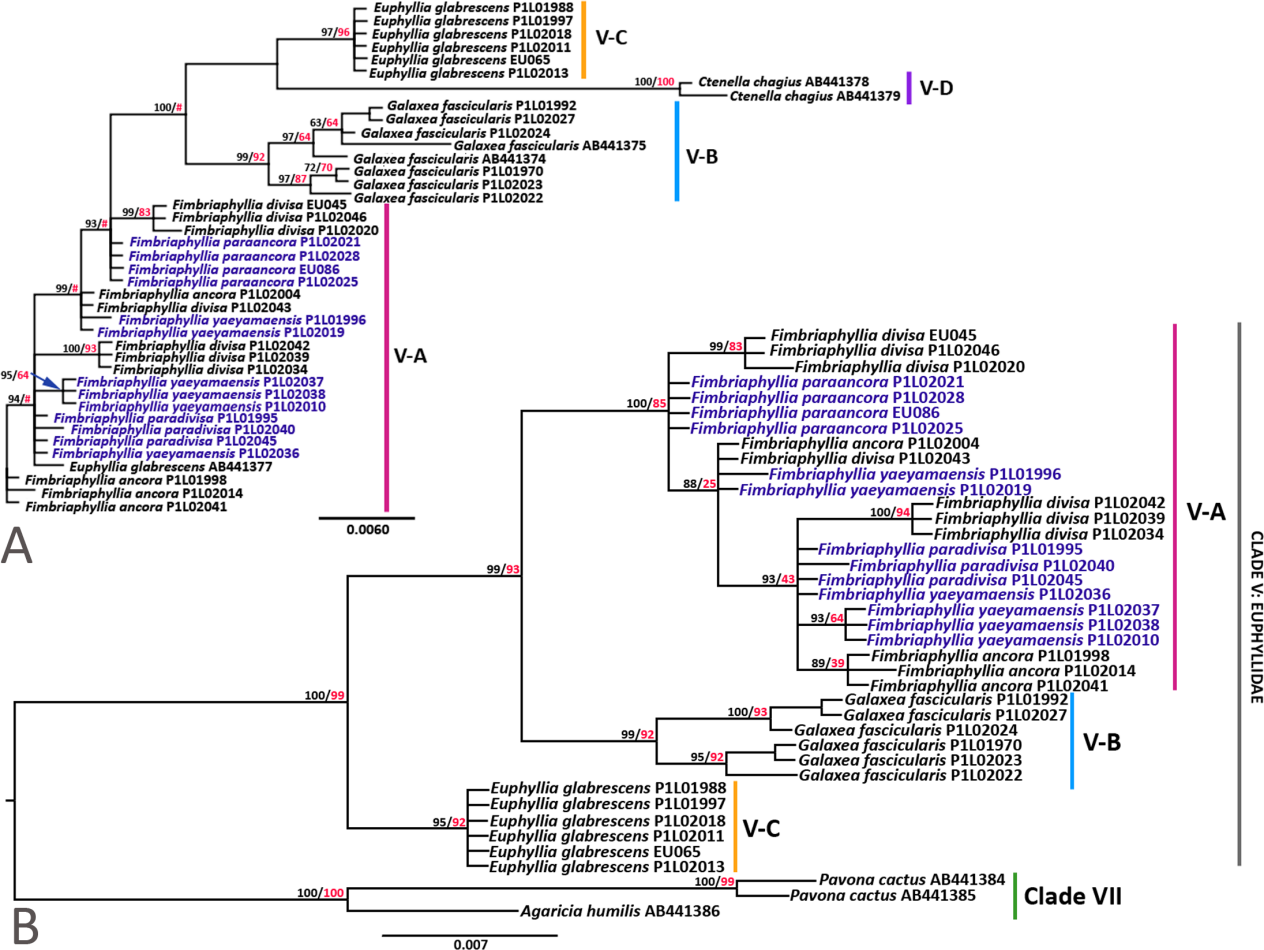
Phylogenetic trees of β-Tubulin. The phylogenetic trees of β-tubulin gene showing (A) the unrooted tree of *Euphyllia, Fimbriaphyllia, Galaxea,* and *Ctenella*; and (B) the gene tree of clade V with clade VII as the outgroup. Bootstrap values for BI (black)/ML (red) are indicated before the nodes of each cluster. # indicates a difference in topologies between the BI and ML gene trees. Species names in blue font were analyzed herein for the first time. Distinct clusters in the tree and the clades are distinguished with vertical lines and labeled accordingly.

Both mitochondrial (*cytb* and *cox1*) and nuclear (β-tubulin) gene trees were congruent in terms of the general topologies, which showed strong statistical support for the clustering of all species of *Euphyllia* with other new members of the family Euphyllidae (*C. chagius* and *G. fascicularis*) (Figs. 1 and 2). Our mitochondrial and β-tubulin sequences were analyzed with the available sequences of *E. ancora* (JF825139 from Lin et al., 2011; AB441289 and AB441290 from Fukami et al., 2008), *E. divisa* (AB441288; Fukami et al., 2008), *E. glabrescens* (ABB441291, ABB441292, and ABB441377; Fukami et al., 2008), *G. fascicularis* (AB441286, AB441287, AB441374, AB441375; Fukami et al., 2008), and *C. chagius* (AB441378 and AB441379; Fukami et al., 2008) from clade V. From a wider context of scleractinian phylogeny, the gene trees of Fukami et al. (2008) consistently showed that clade VI and VII are the closest groups to clade V as they all share the same ancestor (Fig. S1). Sequences of *Pavona cactus* (AB441384 and AB441385 from Fukami et al., 2008), *Pavona clavus* (NC008165; Medina et al., 2006), and *Agaricia humilis* (AB441386 from Fukami et al., 2008; NC008160 from Medina et al., 2006) from clade VII were included to serve as the outgroup for our analyses (Fukami et al., 2008; Lin et al., 2011). A separate phylogenetic tree was generated with the mitochondrial sequences (combined *cox1* and *cytb*) of *Acropora tenuis* (AF338425, NC003522; Van Oppen et al., 2002), *Anacropora matthai* (AY903295, NC006898; Tseng, Wallace & Chen, 2005)*,* and *Montipora cactus* (AY903296, and NC006902; Tseng, Wallace & Chen, 2005) from clade VI (Fig. S2). The combined mitochondrial gene tree generated with clade VII as the outgroup produced the same topology as when clade VI is the outgroup (Fig. S2). Unfortunately, β-tubulin sequences of members of clade VI were not sequenced in the study of Fukami et al. (2008) and are not available at NCBI. Hence, gene trees generated with clade VII will be presented and referred to, mostly, for consistency.

A summary of the gene trees, gene lengths, and the model used to generate the tree is provided in Table S2. All our gene trees exhibited four major groups within the Euphyllidae with the available sequences (Figs. 1 and 2). *Euphyllia ancora, E. divisa, E. paraancora, E. paradivisa,* and *E. yaeyamaensis* were consistently clustered together (group V-A) and diverged from the same ancestor as *G. fascicularis* (group V-B), which formed a separate cluster of its own. All samples of *E. glabrescens* clustered together in group V-C, except for one instance where an *E. glabrescens* sequence (AB441377) clustered under group V-A in the β-tubulin gene tree (Fig. 2A). This may be accounted for by the multi-copy-nature of the β-tubulin gene. It is possible that among the many copies of β-tubulin, the sequence that is AB441377 is an ancient gene copy that may have similarities with the gene sequences of group V-A. Gene introgression poses another possibility; however, this may not be plausible because of the difference in mode of reproduction of *E. glabrescens* and members of group V-A, which will be discussed further later. The cluster of *E. glabrescens* mainly shares the same node with *C. chagius,* but the latter formed a group of its own that is highly divergent from *E. glabrescens* (group V-D).

**Figure 3:**
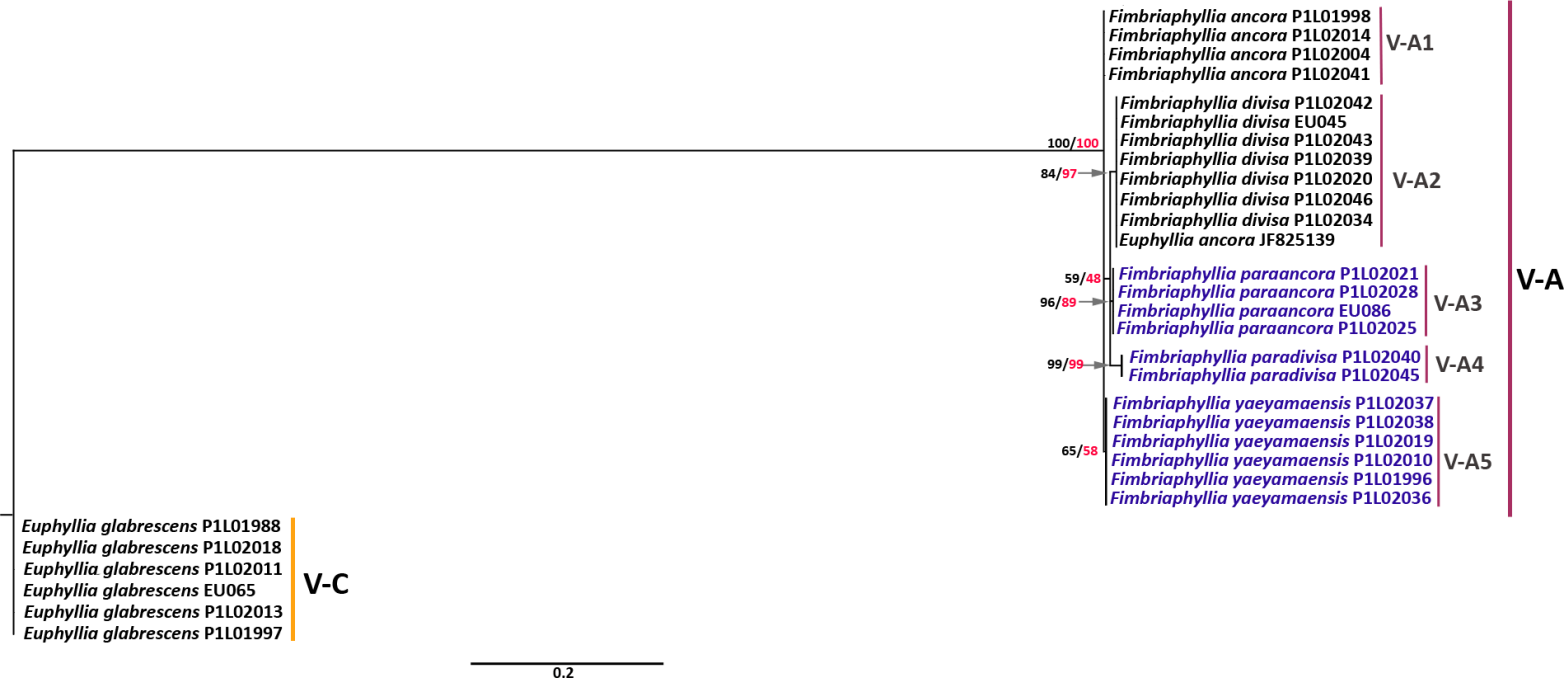
Phylogenetic tree of the 3′-end of the *cox1* gene*.* Bootstrap values of BI (black)/ML (red) trees are indicated before the nodes of the clusters. Major groups in the tree are represented by V-A and V-C. Group V-A has five sub-groups (V-A1 to V-A5). All groups were distinguished with vertical lines and labeled chronologically. Species names in blue font were analyzed herein for the first time.

Among the 22 complete scleractinian mitochondrial genomes examined by Lin et al. (2011), an extra 699 bp at the 3′-end of the *cox1* gene was observed only in the whole mitochondrial genome sequence of *E. ancora* (NC015641). This extra region of the *cox1* gene was also found to occur in all samples of *Euphyllia* in the present study, but was not found in *G. fascicularis* and has not yet been reported in any other member of the Euphyllidae (clade V) as well as in other Scleractinia. Hence, a separate tree was generated for the 3′-end of the *cox1* gene to further examine the internal phylogeny within the genus (Fig. 3). The gene tree generated from the 3′-end of the *cox1* gene showed strong support for two general clusters, groups V-A and V-C, which is congruent with gene trees of β-tubulin, *cytb, cox1,* and the combined mitochondrial genes. More importantly, the gene tree demonstrated finer clustering with high supporting values for five distinct sub-clusters under group V-A. These sub-clusters were identified as *E. ancora* (V-A1)*, E. divisa* (V-A2)*, E. paraancora* (V-A3)*, E. paradivisa* (V-A4)*,* and *E. yaeyamaensis* (V-A5). It was, however, observed that one sample of *E. ancora* (JF825139) grouped in the sub-cluster of *E. divisa* (V-A2)*. Euphyllia ancora* and *E. yaeyamaensis* each have their own distinct group. *Euphyllia divisa, E. paraancora,* and *E. paradivisa,* however, grouped separately from each other under one major cluster.

## Discussion

### Recalling the subgenus *Fimbriaphyllia* and *Euphyllia*

**Figure 4:**
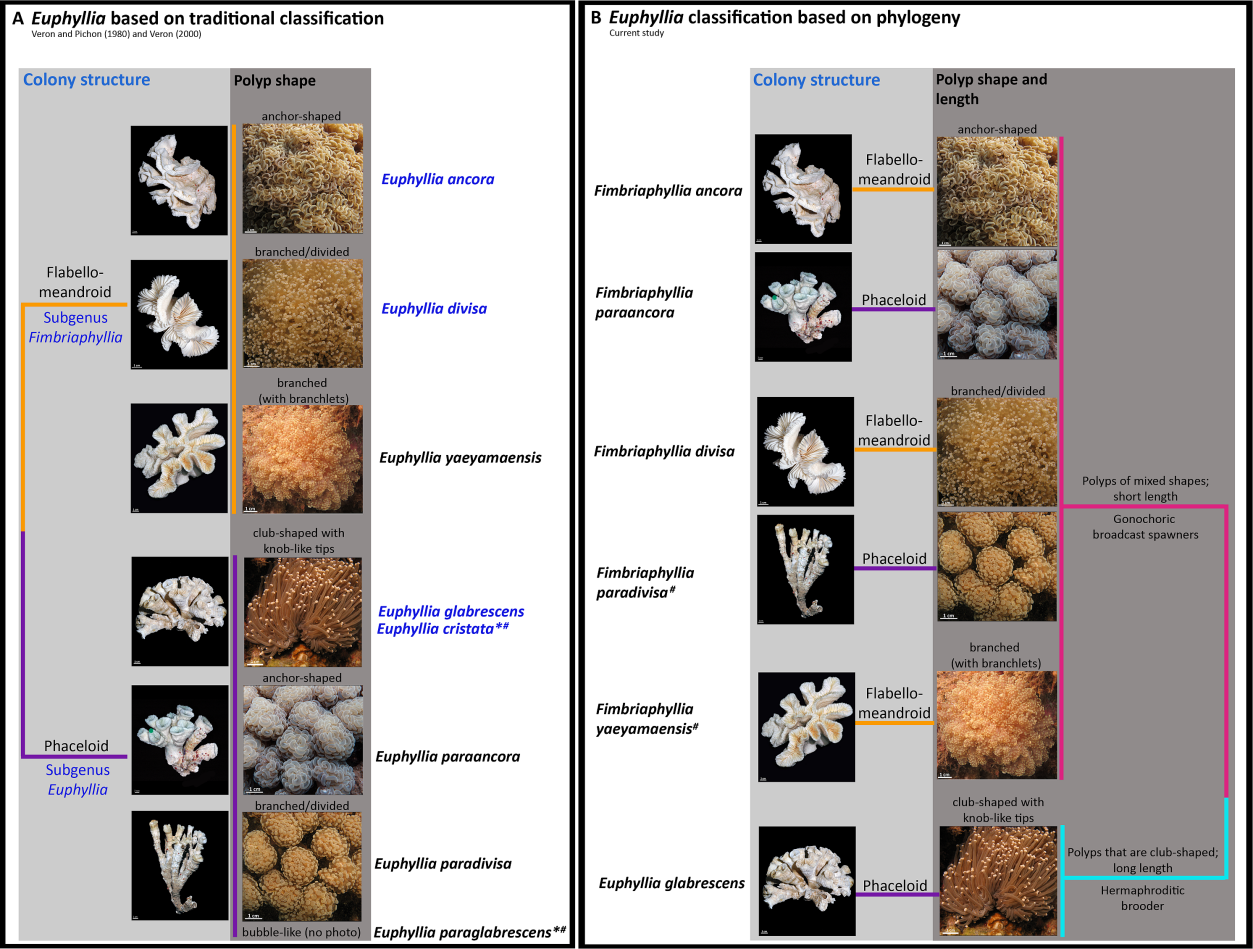
Dichotomous trees of *Euphyllia* and *Fimbriaphyllia.* (A) A dichotomous tree of *Euphyllia* based on Veron & Pichon (1980) and Veron (2000). The species names in blue text are the original members of the subgenera *Euphyllia* and *Fimbriaphyllia* (Veron & Pichon, 1980). The dichotomy corresponds to the phaceloid and flabello-meandroid groups respectively, which Veron (2000) retained even with the synonymy of the subgenera to *Euphyllia* and as new species (in black font) were added to the genus. (B) The phylogenetic-based dichotomous tree, on the other hand, groups species according to polyp morphology and reproductive traits. Photographs of the polyps while fully inflated were taken carefully on field so as not to induce retraction. * species not analyzed in this study. # species with no known records of sexuality or reproductive mode.

Veron & Pichon (1980) classified four *Euphyllia* species into two subgenera under the subfamily Eusmilinae of the family Caryophyllidae. The two subgenera, namely *Fimbriaphyllia* and *Euphyllia,* respectively represented a dichotomy based on colony structure. *E. ancora* and *E. divisa,* being flabello-meandroid in growth form were grouped separately from *E. glabrescens* and *E. cristata,* which had a phaceloid growth form (Fig. 4A, Table 1). Species within a subgenus were identified through polyp shapes as previous classification schemes did not report microstructure characteristics that distinguish between species (Veron & Pichon, 1980; Table 1, Fig. 4A). In all the gene trees, the *Euphyllia* has two distinct paraphyletic groups that are concordant with Fukami et al. (2008) and the phylogenetic tree from the 3′-end of the *cox1* of *Euphyllia* by Lin et al. (2011). Group V-A represents the subgenus *Fimbriaphyllia* with the cluster of *E. ancora* and *E. divisa,* while group V-C represents the subgenus *Euphyllia* with a cluster of *E . glabrescens*. The paraphyly of the two groups with the original members of the subgenera retained in their respective clusters calls for a proposal to elevate the subgenus *Fimbriaphyllia* to genus. Consequentially, the proposal also calls for a revision of the previously established dichotomy between *Euphyllia* and *Fimbriaphyllia,* when they were still subgenera, especially since, based on phylogeny, *Fimbriaphyllia* gained new members. From here on, species in group V-A will bear the genus name *Fimbriaphyllia* when they are referred to in the succeeding discussion*.*

### Revising the dichotomy between *Euphyllia* and *Fimbriaphyllia*

While the subgenera were eventually synonymized as *Euphyllia,* the dichotomy based on colony structure was retained in the field guide of Veron (2000) as new species of *Euphyllia* were discovered and added to the genus (Veron, 1990). The present gene trees for *Euphyllia* still exhibit two major clusters as in the gene trees presented early on by Fukami et al. (2008) and Lin et al. (2011). However, in contrast with the dichotomy of Veron & Pichon (1980) and Veron (2000), the clustering of the phaceloid species of *Fimbriaphyllia paraancora* and *Fimbriaphyllia paradivisa* with the *Fimbriaphyllia* group effectively refutes the dichotomy based on colony structure. This finding is, in part, congruent with Lin et al. (2011), where *F. paraancora* has already been observed to cluster with *E. ancora* and *E. divisa*. With the inclusion of *Euphyllia* species which were not analyzed before, the gene trees now support a dichotomy that is primarily determined by polyp shapes and polyp length instead (Fig. 4B). Furthermore, the phaceloid species, *F. paraancora* (group V-A), *F. paradivisa* (group V-A), and *E. glabrescens* (group V-C), were found to originate from different ancestral nodes in the family, suggesting that the phaceloid colony structure is a convergent trait. The clustering of *Fimbriaphyllia yaeyamaensis,* a species with both phaceloid and flabello-meandroid growth forms, with *Fimbriaphyllia* (group V-A) strengthens the dichotomy based on polyp morphology.

Members of *Fimbriaphyllia* are now characterized with polyps that have projections that are either anchor-shaped or branched (divided). These shapes are the bases of their species names. *Fimbriaphyllia ancora* and *F. paraancora* have anchor-shaped polyps, while *Fimbriaphyllia divisa, F. paradivisa,* and *F. yaeyamaensis* have branched (divided) polyps (Fig. 4B). Polyps in the *Fimbriaphyllia* group are also significantly shorter compared with polyps of the *Euphyllia* group*.* Polyp length, in this study, refers to the observed length of the polyps when they are fully inflated. This means that, polyps of corals from the *Fimbriaphyllia* group are still shorter than the polyps of the *Euphyllia* group even when they are fully inflated (Fig. 4B). Likewise, the length only refers to the regular polyps and not to sweeper tentacles that are, naturally, capable of extending to extreme lengths (i.e., as far as it can extend until it comes in contact with the coral/s beside it) for the purpose of defense. *Euphyllia glabrescens* (group V-C), on the other hand, has polyps that are aptly characterized as long, club-shaped, and glabrous because of the lack of protrusions or projections like branches or branchlets. Among species included in this study, this polyp shape was observed only in *E. glabrescens* and may be diagnostic for group V-C.

Veron & Pichon (1980) classified *E. cristata* under the subgenus *Euphyllia* and Veron (2000) classified *E. paraglabrescens,* together with *E. cristata* and *E. glabrescens,* with the phaceloid group. We were unable to obtain samples of *E. cristata, E. paraglabrescens,* and *E. baliensis* because of their rarity and limited range especially for *E. paraglabrescens* and *E. baliensis,* which are known to occur only in Japan (Veron, 1990) and Indonesia (Turak, Devantier & Erdmann, 2012) respectively. *Euphyllia cristata* has the same polyp shape as *E. glabrescens,* but it is distinct from *E. glabrescens* in having relatively shorter polyps and an exsert primary septa, which can easily be observed when the polyps retract in the field. Given the polyp shape and polyp length of *E. cristata,* we predict from our inferences from the phylogeny presented here that *E. cristata* will cluster under group V-C. As the present study is undergoing review, our prediction has been confirmed with the recently published work of Akmal et al. (2017), that also made use of the same universal primers from Lin et al. (2011), which were also utilized in the present study. *Euphyllia paraglabrescens* and *E. baliensis,* on the other hand, are predicted to cluster with the *Fimbriaphyllia* group on account of having short polyps.

### Morphospecies in the *Euphyllia* and the *Fimbriaphyllia* and the systematic hierarchy

The phylogeny of the 3′-end of the *cox1* gene also supports two major groups (V-A and V-C) that represent the two genera and the dichotomy based on polyp morphology. The distinct clusters or subgroups in the gene tree were found to represent the morphospecies of *Euphyllia* and *Fimbriaphyllia*. In the sub-groupings of the *Fimbriaphyllia* group (sub-groups V-A1 to V-A5 in Fig. 3), species with flabello-meandroid colonies, except for *F. divisa,* clustered separately from each other as in *F. ancora* and *F. yaeyamaensis.* Species with a phaceloid colony structure, as in *F. paraancora* and *F. paradivisa,* each formed their own distinct group, but they were clustered together with the flabello-meandroid, *F. divisa.* In the major cluster of group V-A, *F. yaeyamaensis* and *F. ancora* are at the base of the group, which may mean that the phaceloid species in the group diverged later than the flabello-meandroid species. It appears that species with the same polyp shape have a phaceloid or a flabello-meandroid counterpart. For example, *F. ancora*, a flabello-meandroid species with anchor-shaped polyps, has *F. paraancora* as its phaceloid counterpart. *Fimbriaphyllia yaeyamaensis*, a species with branched polyps, is the only one with both flabello-meandroid and phaceloid colony structures, which makes it unique among species of group V-A. As suggested in the tree, the flabello-meandroid counterpart of *F. yaeyamaensis* with branched polyps is *F. divisa,* and the phaceloid counterpart is *F. paradivisa.* There is the possibility that *Fimbriaphyllia* is still a young group and that introgression may still be occurring among its members; hence, reciprocal monophyly may not have been fully achieved yet. This phenomenon may also explain the grouping of *E. ancora* (JF825139) from Taiwan with *F. divisa* from the Philippines. Nevertheless, among the markers we used, the 3′-end of the *cox1* gene exhibited the best resolution as evidenced by the distinct clusters of species of the *Euphyllia* and the *Fimbriaphyllia* group in the gene tree. Apart from the capability to the resolve species phylogeny in *Euphyllia* and *Fimbriaphyllia,* as predicted by Lin et al. (2011), the ability to tease-out sequences up to the species level and the uniqueness of the gene region to species of these genera shows potential for barcoding. So far, these characteristics are uncommon for the gene markers tested for Scleractinia; furthermore, it was established in the study of Huang et al. (2008) that the mitochondrial gene of anthozoans is slowly evolving and is not suitable for the purposes of DNA barcoding. The 3′-end of the *cox1* gene of *Euphyllia* and *Fimbriaphyllia* may be an exception to the rule.

The phylogeny of the 3′-end of the *cox1* gene shows that it not only supports the dichotomy based on polyp morphology, but it also shows the combination of polyp and skeletal traits that are relevant in distinguishing species of *Fimbriaphyllia* and *Euphyllia* (Fig. 4B). As *E. glabrescens* is the only member of the *Euphyllia* group, so far as our study supports, tubular polyps and phaceloid colony structure is a combination of traits that have been found to be unique to the species. *Euphyllia cristata* shares the same combination of traits but its septal morphology has been described to be distinct from *E. glabrescens* (Veron & Pichon, 1980; Table 1). The finer clustering supporting the morphospecies and the distinction between *E. glabrescens* and *E. cristata* at the miscrostructure level opens the possibility that there may be microstructures that may also be able to diagnose species of *Fimbriaphyllia*.

### External relationships of the *Euphyllia* and *Fimbriaphyllia* with the new members of Euphyllidae (clade V)

Veron & Pichon (1980) and Veron (2000) described features of the Euphylliidae that we found to be shared features with *G. fascicularis* and *C. chagius*. The euphylliids, *G. fascicularis,* and *C. chagius,* are usually dome-shaped and massive, and yet coralla are light-weight with a blistery coenosteum. Colony structures of the Euphyllidae include phaceloid, flabello-meandroid, and meandroid. Septa are round or lobe-shaped with granulated or glabrous sides and margins. Walls are septothecal or parathecal. *Galaxea fascicularis* (Veron & Pichon, 1980) and *C. chagius* (Sheppard, Dinesen & Drew, 1983) also have granulated septa with variable costae that may be striated or ornamented with lobes or spines.

The clustering of *G. fascicularis* and *C. chagius* under the family Euphyllidae might not be surprising to some systematists. Previous classification schemes and species descriptions already suggested that *Galaxea* and *Ctenella* be grouped with *Euphyllia.* In the family tree of Scleractinia, Veron (2000) showed that the family Euphyllidae diverged from the family Oculinidae. In the same family tree, Oculinidae was shown to be grouped with the family Meandrinidae under the suborder Meandrina, where *C. chagius* was classified. Vaughan & Wells (1943) described *Euphyllia* and *Ctenella* together under the subfamily Eusmilinae of the Caryophyllidae (suborder Caryophyllida) on the bases of having exsert septa, intratentacular budding, septothecal walls, and a brown polyp color. Sheppard, Dinesen & Drew (1983) also suggested a revision of Meandrinidae stating that *Ctenella* be grouped under the Eusmilinae on the basis of having “smooth septal margins and a light-weight coralla”. *Galaxea fascicularis* and *C. chagius* also have fleshy polyps that are usually extended during daytime. Sweeper tentacles and extracoelomic digestion were also documented in *Euphyllia, Galaxea,* and *C. chagius* (Sheppard, Dinesen & Drew, 1983; Hidaka, 1985; Borneman, 2001). While *C. chagius* is meandroid, which is already a known trait of the Euphyllidae, the inclusion of *Galaxea* adds a plocoid growth form to the family (clade V).

The affinity of *G. fascicularis* and *C. chagius* with euphyllids is, so far, consistent with the dichotomy based on polyp morphology. *Galaxea fascicularis* has short polyps as in species of *Fimbriaphyllia*, while *C. chagius* has the same long and tubular polyps as in *E. glabrescens.* Despite the shared characteristics of *C. chagius* with the Euphyllidae, *C. chagius* is highly divergent in the Euphyllidae (clade V). This divergence may indicate differences in the skeletal morphology of *C. chagius* with *Euphyllia* and *Fimbriaphyllia*. *Galaxea fascicularis, Euphyllia,* and *Fimbriaphyllia* species usually have three to four or sometimes five orders of septa and the columella is often weakly developed or absent (Veron & Pichon, 1980; Veron, 1990; Veron, 2000). *Ctenella chagius,* on the other hand, is characterized by two orders of septa and the presence of a lamellar or continuous columella (Sheppard, Dinesen & Drew, 1983). The high divergence may also be attributed to the limited geographical range of *C. chagius* as it has only been reported to occur locally in the Chagos Archipelago of the Indian Ocean (Sheppard, Dinesen & Drew, 1983). There have been no new records of the species elsewhere to date (Sheppard, Turak & Wood, 2008; Carpenter et al., 2008). In relation to having restricted species ranges, the systematic positions of *Gyrosmilia* and *Montigyra,* monotypic genera of the family Meandrinidae that are found only in the Indian Ocean, have not yet been analyzed from the molecular perspective. All our samples of *Euphyllia, Fimbriaphyllia,* and *Galaxea* are from the Pacific Ocean, but their geographic ranges extend until the Indian Ocean (Veron, 2000). Hence, the inclusion of samples of euphylliids from the Indian Ocean may strengthen the phylogeny presented here. On the other hand, there is a possibility that Indian Ocean specimens may also show divergence between populations of the same species collected from the Pacific and Indian Oceans (Fukami et al., 2004) as in *Favites complanata, Favia rotumana,* and *Favia pallida* (clade XVII) (Arrigoni et al., 2012).

### Evolution of reproductive traits in the Euphyllidae (clade V)

Sexuality and modes of reproduction are emergent patterns that were also perceived in the clustering in the gene trees. *Fimbriaphyllia ancora*, *F. divisa,* and *F. paraancora* (group V-A)*,* are mainly gonochoric (dioecious) broadcast spawners (Borneman, 2003; Twan, Hwang & Chang, 2003; Twan et al., 2005; Twan et al., 2006; Fan et al., 2006). To date, there is no scientifically published information about the reproductive mode of *F. paradivisa* and *F. yaeyamaensis,* but we predict that they are also gonochoric broadcast-spawners as in other species of *Fimbriaphyllia*. *Euphyllia glabrescens* (group V-C)*,* on the other hand, is known to be a hermaphroditic brooder (Richmond & Hunter, 1990; Baird, Guest & Willis, 2009). Sexuality and reproductive modes are still unknown for *E. cristata, E. paraglabrescens,* and *E. baliensis. Galaxea fascicularis* was first reported to be a hermaphroditic broadcast-spawner (Babcock et al., 1986; Shlesinger, Goulet & Loya, 1998) but was later reported to be a pseudo-gynodioecious broadcast-spawner instead (Guest et al., 2005; Baird, Guest & Willis, 2009). Keshavmurthy et al. (2012) were able to resolve the sexuality of the species by showing that *G. fascicularis* is gynodioecious rather than pseudo-gynodioecious. Being gynodioecious is characterized by the existence of female colonies separate from hermaphroditic colonies that produce viable egg and sperm (Keshavmurthy et al., 2012). Pseudo-gynodioecious, on the other hand, is essentially the same, but hermaphroditic colonies are thought to produce non-viable eggs, hence “pseudo” (Guest et al., 2005; Baird, Guest & Willis, 2009). The gynodioecious type of sexuality has not yet been documented in other corals and is, to date, unique to *G. fascicularis* (Keshavmurthy et al., 2012). The sexuality of *G. fascicularis,* being gynodioecious, appears to be an intermediate or a “transitional state” between *Fimbriaphyllia* (dioecious) and *Euphyllia* (hermaphroditic), which was also mentioned by Keshavmurthy et al. (2012). This claim is supported by our gene trees, and it may explain why *G. fascicularis* is in its own cluster, but its close affinity to *Fimbriaphyllia* is based on being a broadcast-spawner. Unfortunately, the sexuality and mode of reproduction of *C. chagius* has not yet been documented, but as the pattern in our gene tree suggests, *C. chagius* is hypothesized to be a hermaphroditic brooder like *E. glabrescens*.

The gene trees of Fukami et al. (2008) clustered *Euphyllia, Galaxea,* and *Ctenella,* which are now new members of the family Euphyllidae (clade V; Fukami et al., 2008). The general topology in our gene trees is concordant with the gene trees of Fukami et al. (2008) and Lin et al. (2011) even with the inclusion of *F. yaeyamaensis, F. paraancora,* and *F. paradivisa*. The inclusion of other species of *Euphyllia* in the phylogenetic reconstruction of the Euphyllidae led to the recognition of the former subgenera in the *Euphyllia,* which called for a proposal to recall and elevate *Fimbriaphyllia* as a genus. The phylogeny of *Euphyllia* and the *Fimbriaphyllia* shows that systematics need not be limited to skeletal traits and the distinction between morphospecies highlights the importance of combining polyp morphology and reproductive traits with skeletal morphology, which are of higher systematic value.

## Conclusions and Recommendations

The presence of two distinct and well-supported groups of *Euphyllia* in all the gene trees that retained the original members of the previous subgenera of *Euphyllia* and *Fimbriaphyllia* supports the recognition of *Fimbriaphyllia* as a genus. While the two subgenera previously represented a dichotomy that is based on colony structure, the dichotomy between the genera of *Euphyllia* and *Fimbriaphyllia* is now defined by polyp shapes, polyp length, sexuality, and mode of reproduction. The finer clustering of the 3′-end of the *cox1* gene exhibiting the distinct morphospecies of *Euphyllia* and *Fimbriaphyllia* shows that species are best identified through combining polyp morphology and colony structure. In the *Euphyllia* group, septal morphology appears to distinguish between species; thus, opening the possibility that there may be microstructures of the skeleton that may also be able to distinguish between species of *Fimbriaphyllia*.

The morphological characteristics and other biological attributes that were identified through the integrated approach on the, previously, largest genus in the Euphyllidae (clade V) have shown patterns that are consistent with the other members of the clade as in *G. fascicularis* and *C. chagius* each diverged from the same ancestral node as *Fimbriaphyllia* and *Euphyllia* respectively*.* Apart from some similarities in skeletal traits, the polyp features that were identified for *Fimbriaphyllia* and *Euphyllia* were also found to be applicable to *G. fascicularis* and *C. chagius*. The pseudo-gynodioecious broadcast spawning trait of *G. fascicularis,* which was previously hypothesized as a transitional state between *Fimbriaphyllia* and *Euphyllia*, has been confirmed in this study. These findings show that the phylogenetic-based systematic scheme of *Euphyllia, Fimbriaphyllia, Galaxea,* and *Ctenella* are potentially operationally useful for the rest of clade V. Thus, laying the groundwork for fully resolving the phylogeny of the family Euphyllidae (clade V) as there are still species that still lack in molecular analyses. These species include two more species of *Euphyllia*, namely *E. paraglabrescens* and *E. baliensis,* which are of limited biogeographic range. *Gyrosmilia interrupta* and *Montigyra kenti*, species from the same family as *C. chagius* and also limited to the Indian Ocean, are also still unresolved. The inclusion of species of *Euphyllia, Fimbriaphyllia,* and *Galaxea* that are also found in the Indian Ocean and are within the geographic range of *C. chagius* is recommended for future studies.

##  Supplemental Information

10.7717/peerj.4074/supp-1 Table S1 List of specimen collection *Euphyllia* and *Galaxea* specimens collected from the Philippines (Talim Bay, Lian, Batangas and Bolinao, Pangasinan) and Taiwan and their corresponding Accession numbers in GenBank. Coralla are kept in the Coral Museum of the University of the Philippines—The Marine Science Institute (UP-MSI), while the DNA is kept at −80 °C at the Molecular Science Unit—De La Salle University, Philippines (DLSU) and in Academia Sinica, Taiwan (AST).
*No corallum specimen left after the tissue collection but the DNA has been archived. The DNA number is provided instead.

10.7717/peerj.4074/supp-2 Table S2 Genes and alignment schemes Genes and alignment schemes with their corresponding base pair sizes and models of evolution that were used to generate gene trees using BI and ML.

10.7717/peerj.4074/supp-3 Figure S1 Scleractinian phylogenetic tree of Fukami et al. (2008) A simplified illustration of the scleractinian phylogenetic tree of Fukami et al. (2008), which highlights (in yellow) the position of *Euphyllia* and clade V relative to the complex clade (outlined in blue-green), the robust clade (outlined in red), and the outgroups (Zoanthidea, Actiniaria, Antipatharia, and Corallimorpharia; outlined in gray). Clades VI and VII (highlighted in green) of the complex clade (outlined in blue-green) are the outgroups of clade V in the present study.

10.7717/peerj.4074/supp-4 Figure S2 Phylogenetic tree of combined *cox1* and *cytb* genes with sequences from clade VI as an outgroup** The phylogenetic tree of the combined *cox1* and *cytb* of species in clade V and with clade VI as an outgroup.** Bootstrap values of BI (black)/ML (red) are indicated before the nodes of the clusters. # indicates a difference in topologies between the BI and ML gene trees. Species names in blue font were analyzed herein for the first time. Distinct clusters in the tree and the clades are distinguished with vertical lines and labeled accordingly.

## Additional Information and Declarations

### Field Study Permissions

The following information was supplied relating to field study approvals (i.e., approving body and any reference numbers): A gratuitous permit to collect scleractinian corals was approved by the Bureau of Fisheries and Aquatic Resources (BFAR) of the Philippines. The corals in Taiwan were collected through the permission granted by the Kenting National Park Authority as part of a long-term ecological monitoring program.

### DNA Deposition

The following information was supplied regarding the deposition of DNA sequences: All DNA sequences in this study are accessible via GenBank®. Please see the Supplemental File (Table S2) for the accession numbers.

### Data Availability

The following information was supplied regarding data availability: GenBank-Please see the supplemental file provided (Table S2).
