# Correction: Resurrecting a subgenus to genus: molecular phylogeny of *Euphyllia* and *Fimbriaphyllia* (order Scleractinia; family Euphylliidae; clade V)

**DOI:** 10.7717/peerj.4074/correction-1

**Published:** 2018-07-08

**Authors:** Katrina S. Luzon, Mei-Fang Lin, Ma. Carmen A. Ablan Lagman, Wilfredo Roehl Y. Licuanan, Chaolun Allen Chen

**Affiliations:** 1 Biology Department, De La Salle University, Manila, Philippines; 2 Shields Ocean Research (SHORE) Center, De La Salle University, Manila, Philippines; 3 The Marine Science Institute, University of the Philippines, Quezon City, Philippines; 4 Biodiversity Research Center, Academia Sinica, Taipei, Taiwan; 5 Department of Molecular and Cell Biology, James Cook University, Townsville, Australia; 6 Evolutionary Neurobiology Unit, Okinawa Institute of Science and Technology Graduate University, Okinawa, Japan; 7 Center for Natural Sciences and Environmental Research (CENSER), De La Salle University, Manila, Philippines; 8 Taiwan International Graduate Program-Biodiversity, Academia Sinica, Taipei, Taiwan; 9 Institute of Oceanography, National Taiwan University, Taipei, Taiwan

Corrigendum: 

After publication, a reader notified the authors that there are taxonomic and spelling issues throughout the article and in the reference list. The following changes have been made: 

A type species has been identified for Fimbriaphyllia, which is now assigned to the family Euphylliidae. The publication is now registered with ZooBank (urn:lsid:zoobank.org:pub:EBF69BA0-897E-4AC8-ADF5-4A7115CA1353).

Physogyra lichtensteni, Plerogyra sinuosa , Plesiastrea versipora and Blastomussa wellsi are now classified as incertae sedis.

Faviidae is no longer listed as a family. Favia rotumana is now referred to as Dipsastraea rotumana, and Favia pallida is now referred to as Dipsastraea pallida.

The taxonomic status of Trachyphyllidae [sic] has been removed.

F. divisa is now described as having both flabello-meandroid and phacelo-flabellate or phaceloid corallite structures.

Euphyllidae is now spelled as Euphylliidae, Caryophyllidae as Caryophylliidae and Lobophyllidae as Lobophylliidae.

Figure 4 has been revised to reflect that Fimbriaphyllia divisa and Fimbriaphyllia yaeyamaensis both have phaceloid and phacelo-flabellate colony formations. These traits are indicated in the branches next to the photos of F. divisa and F. yaeyamaensis in the dichotomous tree based on phylogeny.

New Figures 5 and 6 have been added to demonstrate the skeletal morphology of the Euphyllia and Fimbriaphyllia species, and height comparisons between F. paradivisa and E. glabrescens, respectively.

New references Alloiteau (1952), Benzoni et al. (2014), Budd et al. (2012), Chamisso & Eysenhardt (1821), Chen et al. (2005), Eyal et al. (2016), Gravier (1910), Griffith (2004), Huang et al. (2016), Khodzori et al. (2015), Matthai (1928), Mazlan et al. (2005), Milne & Haime (1848), Milne & Haime (1849), Milne & Haime (1851), Nemenzo (1960), Oken (1815-1816), Pillai (1971), Quoy & Gaimard (1824), Richards & Beger (2013), Saville-Kent (1893), Shirai (1980), Spengler (1799), Umbgrove (1939), Veron & Hodgson (1989), Veron (1990), Veron (1992) and Wells (1971) have been added. 

Additionally, titles for references Akmal et al. (2017), Arrigoni et al. (2016) and Veron & Pichon (1980) have been corrected.

Lastly, the authors have added an Acknowledgements and a New Species Registration section and have updated the Funding statement, Grant Disclosure, and Field Study Permissions to reflect additional work done post-publication. 

Publisher’s Note: On July 8, 2018, this corrigendum was published with an incomplete LSID. This has now been corrected to: urn:lsid:zoobank.org:pub:EBF69BA0-897E-4AC8-ADF5-4A7115CA1353

