# Supplementary material for: Resurrecting a subgenus to genus: molecular phylogeny of Euphyllia and Fimbriaphyllia (order Scleractinia; family Euphyllidae; clade V)
Source: PeerJ. 2017 Dec 4;5:e4074. doi: 10.7717/peerj.4074 (PMC5719963; doi:10.7717/peerj.4074)
Supplement: Table S1 — Euphyllia and Galaxea specimens collected from the Philippines (Talim Bay, Lian, Batangas and Bolinao, Pangasinan) and Taiwan and their corresponding Accession numbers in GenBank. Coralla are kept in the Coral Museum of the University of the Philippines—The Marine Science Institute (UP-MSI), while the DNA is kept at −80 °C at the Molecular Science Unit—De La Salle University, Philippines (DLSU) and in Academia Sinica, Taiwan (AST). *No corallum specimen left after the tissue collection but the DNA has been archived. The DNA number is provided instead. [file peerj-05-4074-s001.docx]

| Species name | Voucher number of Tissue | Museum Specimen Number | GenBank Accession Numbers | | | Place of collection | Corallum/DNA storage location |
| --- | --- | --- | --- | --- | --- | --- | --- |
|  |  |  | COX1 | CYTB | Β-tubulin |  |  |
| *Euhyllia ancora* | EU028 | P1L01998 | KU233261 | KU233329 | KU233293 | Lian, Batangas, Philippines | UPMSI/ DLSU and AST |
| *Euphyllia ancora* | EU076 | P1L02014 | KU233262 | KU233330 | KU233294 | Lian, Batangas, Philippines | UPMSI/ DLSU and AST |
| *Euphyllia ancora* | EU077 | P1L02004 | KU233263 | KU233331 | KU233295 | Lian, Batangas, Philippines | UPMSI/ DLSU and AST |
| *Euphyllia ancora* | EU078 | P1L02041 | KU233264 | KU233332 | KU233296 | Lian, Batangas, Philippines | UPMSI/ DLSU and AST |
| *Euphyllia divisa* | EU042 | P1L02042 | KU233265 | KU233333 | KU233297 | Lian, Batangas, Philippines | UPMSI/ DLSU and AST |
| *Euphyllia divisa* | EU045 | ** | KU233266 | KU233334 | KU233298 | Lian, Batangas, Philippines | -----/ DLSU and AST |
| *Euphyllia divisa* | EU050 | P1L02043 | KU233267 | KU233335 | KU233299 | Lian, Batangas, Philippines | UPMSI/ DLSU and AST |
| *Euphyllia divisa* | EU051 | P1L02039 | KU233268 | KU233336 | KU233300 | Lian, Batangas, Philippines | UPMSI/ DLSU and AST |
| *Euphyllia divisa* | EU052 | P1L02046 | KU233269 | KU233337 | KU233301 | Lian, Batangas, Philippines | UPMSI/ DLSU and AST |
| *Euphyllia divisa* | EU053 | P1L02034 | KU233270 | KU233338 | KU233302 | Lian, Batangas, Philippines | UPMSI/ DLSU and AST |
| *Euphyllia divisa* | EU063 | P1L02020 | KU233271 | KU233339 | KU233303 | Lian, Batangas, Philippines | UPMSI/ DLSU and AST |
| *Euphyllia yaeyamaensis* | EU027 | P1L01996 | KU233272 | KU233346 | KU233317 | Lian, Batangas, Philippines | UPMSI/ DLSU and AST |
| *Euphyllia yaeyamaensis* | EU055 | P1L02036 | KU233273 | KU233347 | KU233318 | Lian, Batangas, Philippines | UPMSI/ DLSU and AST |
| *Euphyllia yaeyamaensis* | EU056 | P1L02037 | KU233274 | KU233348 | KU233319 | Lian, Batangas, Philippines | UPMSI/ DLSU and AST |
| *Euphyllia yaeyamaensis* | EU057 | P1L02038 | KU233275 | KU233349 | KU233320 | Lian, Batangas, Philippines | UPMSI/ DLSU and AST |
| *Euphyllia yaeyamaensis* | EU058 | P1L02019 | KU233276 | KU233350 | KU233321 | Lian, Batangas, Philippines | UPMSI/ DLSU and AST |
| *Euphyllia yaeyamaensis* | EU075 | P1L02010 | KU233277 | KU233351 | KU233322 | Lian, Batangas, Philippines | UPMSI/ DLSU and AST |
| *Euphyllia paradivisa* | EU012 | P1L01995 | -------------- | -------------- | KU233314 | Lian, Batangas, Philippines | UPMSI/ DLSU and AST |
| *Euphyllia paradivisa* | EU041 | P1L02040 | KU233278 | KU233344 | KU233315 | Lian, Batangas, Philippines | UPMSI/ DLSU and AST |
| *Euphyllia paradivisa* | EU054 | P1L02045 | KU233279 | KU233345 | KU233316 | Lian, Batangas, Philippines | UPMSI/ DLSU and AST |
| *Euphyllia paraancora* | EU084 | P1L02021 | KU233280 | KU233340 | KU233310 | Bolinao, Pangasinan, Philippines | UPMSI/ DLSU and AST |
| *Euphyllia paraancora* | EU085 | P1L02028 | KU233281 | KU233341 | KU233311 | Bolinao, Pangasinan, Philippines | UPMSI/ DLSU and AST |
| *Euphyllia paraancora* | EU086 | ** | KU233282 | KU233342 | KU233312 | Bolinao, Pangasinan, Philippines | -----/ DLSU and AST |
| *Euphyllia paraancora* | EU087 | P1L02025 | KU233283 | KU233343 | KU233313 | Bolinao, Pangasinan, Philippines | UPMSI/ DLSU and AST |
| *Euphyllia glabrescens* | EU010 | P1L01988 | KU233284 | KU233352 | KU233304 | Lian, Batangas, Philippines | UPMSI/ DLSU and AST |
| *Euphyllia glabrescens* | EU026 | P1L01997 | KU233285 | ------------- | KU233305 | Lian, Batangas, Philippines | UPMSI/ DLSU and AST |
| *Euphyllia glabrescens* | EU061 | P1L02018 | KU233286 | KU233353 | KU233306 | Lian, Batangas, Philippines | UPMSI/ DLSU and AST |
| *Euphyllia glabrescens* | EU064 | P1L02011 | KU233287 | KU233354 | KU233307 | Lian, Batangas, Philippines | UPMSI/ DLSU and AST |
| *Euphyllia glabrescens* | EU065 | ** | KU233288 | -------------- | KU233308 | Kenting National Park, Taiwan | UPMSI/ DLSU and AST |
| *Euphyllia glabrescens* | EU066 | P1L02013 | KU233289 | KU233355 | KU233309 | Lian, Batangas, Philippines | UPMSI/ DLSU and AST |
| *Galaxea fascicularis* | GA014 | P1L01992 | KU233290 | KU233356 | KU233323 | Lian, Batangas, Philippines | UPMSI/ DLSU and AST |
| *Galaxea fascicularis* | GA016 | P1L01970 | KU233291 | KU233357 | KU233324 | Lian, Batangas, Philippines | UPMSI/ DLSU and AST |
| *Galaxea fascicularis* | GA046 | P1L02027 | KU233292 | KU233358 | KU233325 | Lian, Batangas, Philippines | UPMSI/ DLSU and AST |
| *Galaxea fascicularis* | GA047 | P1L02022 | -------------- | KU233359 | KU233326 | Lian, Batangas, Philippines | UPMSI/ DLSU and AST |
| *Galaxea fascicularis* | GA048 | P1L02023 | -------------- | KU233360 | KU233327 | Lian, Batangas, Philippines | UPMSI/ DLSU and AST |
| *Galaxea fascicularis* | GA049 | P1L02024 | -------------- | KU233361 | KU233328 | Lian, Batangas, Philippines | UPMSI/ DLSU and AST |
