# Supplementary material for: Resurrecting a subgenus to genus: molecular phylogeny of Euphyllia and Fimbriaphyllia (order Scleractinia; family Euphyllidae; clade V)
Source: PeerJ. 2017 Dec 4;5:e4074. doi: 10.7717/peerj.4074 (PMC5719963; doi:10.7717/peerj.4074)
Supplement: Table S2 — Genes and alignment schemes with their corresponding base pair sizes and models of evolution that were used to generate gene trees using BI and ML. [file peerj-05-4074-s002.docx]

| **Gene Marker** | **Alignment Scheme** | | **Total number of sites** | **Model of Evolution** |
| --- | --- | --- | --- | --- |
| Combined *cytb* and *cox1* | clade V with clade VII | | 1428 | HKY+G |
|  | clade V with clade VI | | 1428 | HKY |
| *Cytb* | *Euphyllia* sequences with *Galaxea* | | 774 | HKY |
| *Cox1* | 3’-end of *Euphyllia* | | 948 | HKY |
|  | *Euphyllia* with *Galaxea* | | 825 | HKY |
| β-tubulin | | clade V | 442 | K2+G |
|  |  | clade V with clade VII | 442 | K2+G |
