# Supplementary figures and images for: Resurrecting a subgenus to genus: molecular phylogeny of Euphyllia and Fimbriaphyllia (order Scleractinia; family Euphyllidae; clade V)
Source: PeerJ. 2017 Dec 4;5:e4074. doi: 10.7717/peerj.4074 (PMC5719963; doi:10.7717/peerj.4074)

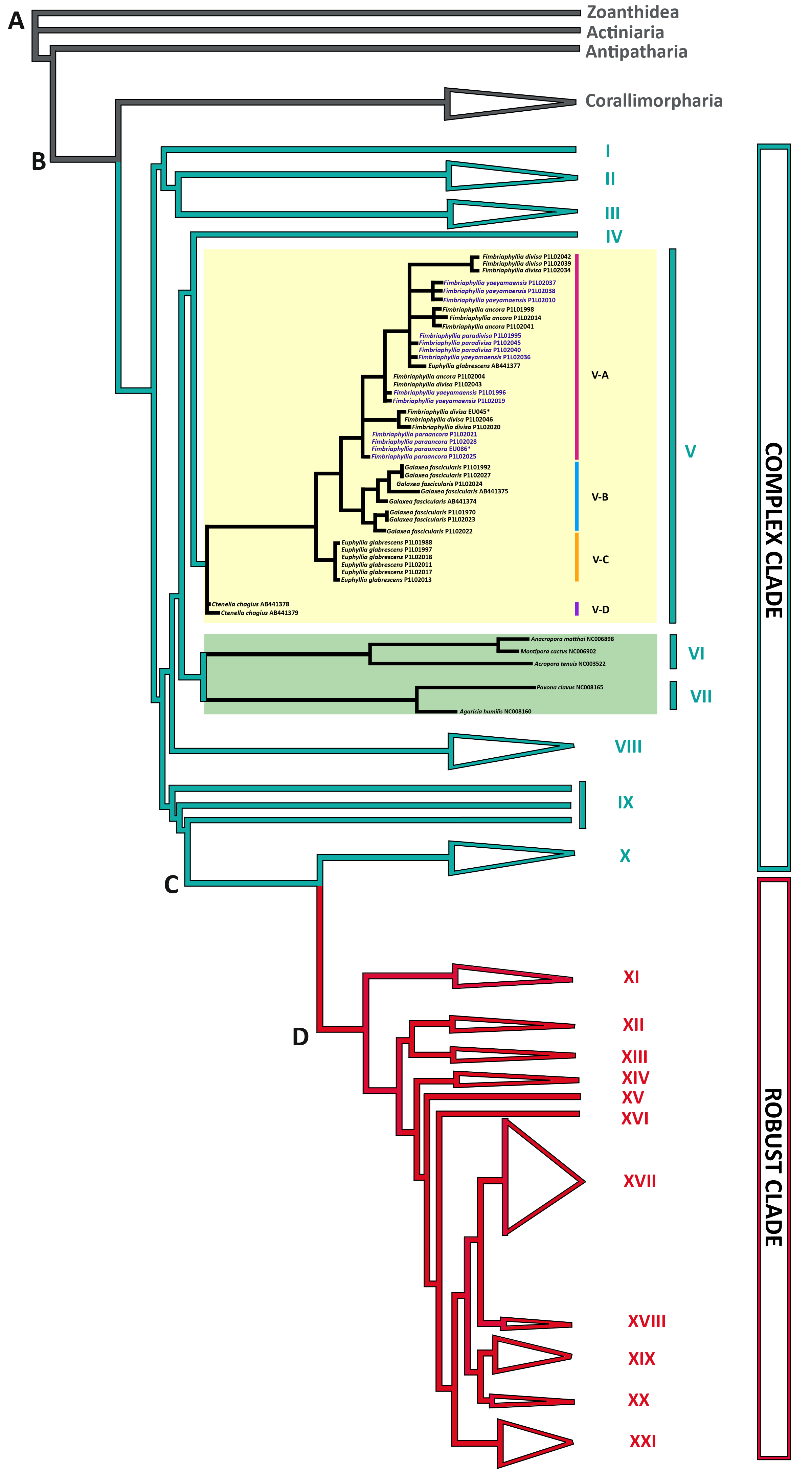

Supplement: Figure S1 — A simplified illustration of the scleractinian phylogenetic tree of Fukami et al. (2008), which highlights (in yellow) the position of Euphyllia and clade V relative to the complex clade (outlined in blue-green), the robust clade (outlined in red), and the outgroups (Zoanthidea, Actiniaria, Antipatharia, and Corallimorpharia; outlined in gray). Clades VI and VII (highlighted in green) of the complex clade (outlined in blue-green) are the outgroups of clade V in the present study. [file peerj-05-4074-s003.png]

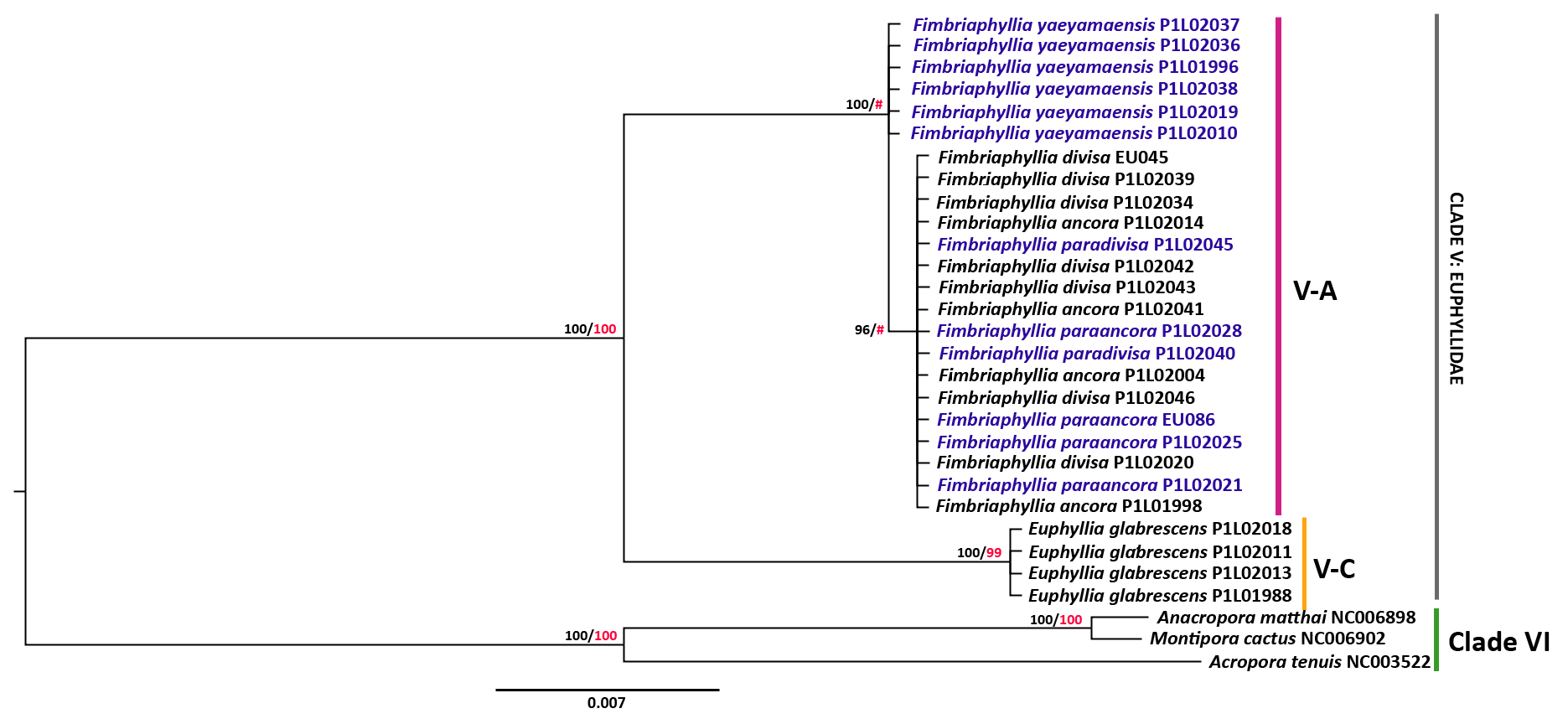

Supplement: Figure S2 — The phylogenetic tree of the combined cox1 and cytb of species in clade V and with clade VI as an outgroup. Bootstrap values of BI (black)/ML (red) are indicated before the nodes of the clusters. # indicates a difference in topologies between the BI and ML gene trees. Species names in blue font were analyzed herein for the first time. Distinct clusters in the tree and the clades are distinguished with vertical lines and labeled accordingly. [file peerj-05-4074-s004.png]
